# Exploring the association between dietary indices and metabolic dysfunction-associated steatotic liver disease: Mediation analysis and evidence from NHANES

**DOI:** 10.1371/journal.pone.0321251

**Published:** 2025-04-17

**Authors:** Qiang Wang, Rude Chen, Shaohua Chen, Bowen Wei, Chunlan Liu, Zongxing Jiang

**Affiliations:** Xindu District People’s Hospital of Chengdu, Chengdu, Sichuan, China; Mayo Clinic Florida: Mayo Clinic's Campus in Florida, UNITED STATES OF AMERICA

## Abstract

**Background:**

The association between dietary indices and metabolic dysfunction-associated steatotic liver disease (MASLD) has shown inconsistent results in previous studies. Additionally, the potential mediating variables linking dietary quality to MASLD have not been adequately explored.

**Methods:**

We analyzed data from 6,369 participants in the National Health and Nutrition Examination Survey (NHANES) 2007–2018. Three dietary indices—Healthy Eating Index (HEI), Energy-adjusted Dietary Inflammatory Index (EDII), and Composite Dietary Antioxidant Index (CDAI)—were evaluated for their associations with MASLD using logistic regression models adjusted for a comprehensive range of covariates. Mediation analysis was performed to evaluate the roles of potential mediators from four domains: insulin resistance (homeostatic model assessment of insulin resistance, HOMA-IR; metabolic score for insulin resistance, METS-IR), systemic inflammation (systemic inflammatory response index, SIRI; systemic immune-inflammation index, SII), obesity or visceral fat distribution (a body shape index, ABSI; body roundness index, BRI), and oxidative stress (Gamma-Glutamyltransferase, GGT; Bilirubin; Uric Acid).

**Results:**

After adjusting for all covariates, only HEI showed a consistent inverse association with MASLD, while EDII and CDAI showed no significant associations. Mediation analysis identified METS-IR, HOMA-IR, BRI, and ABSI as significant mediators in the relationship between HEI and MASLD, with mediation proportion accounting for 47.16%, 48.84%, 52.69%, and 13.84%, respectively.

**Conclusion:**

Higher HEI is associated with a reduced risk of MASLD. The findings suggest that insulin resistance and visceral fat distribution partially mediate the relationship between HEI and MASLD, providing insights into potential mechanisms linking diet and liver health.

## 1. Introduction

Metabolic dysfunction-associated steatotic liver disease (MASLD), previously known as non-alcoholic fatty liver disease (NAFLD), is now recognized as a significant global health concern [1]. It is defined by abnormal fat deposition in liver cells in the absence of substantial alcohol intake or other identifiable liver disease causes [2]. MASLD is intricately linked to metabolic abnormalities, including insulin resistance, type 2 diabetes mellitus, obesity, dyslipidemia, and hypertension [3,4]. The rising prevalence of MASLD reflects the worldwide increase in obesity and metabolic health challenges, making it a pressing concern for healthcare systems worldwide [5]. Understanding the modifiable factors that influence MASLD risk is critical for developing effective prevention and intervention strategies.

Dietary patterns and nutritional quality are key modifiable factors in the prevention and management of MASLD [6]. High-quality diets have been associated with improved metabolic health outcomes, whereas diets rich in saturated fats, processed foods, and added sugars are linked to increased risk of metabolic dysfunction [6,7]. Dietary indices, such as the Healthy Eating Index (HEI), Energy-adjusted Dietary Inflammatory Index (EDII), and Composite Dietary Antioxidant Index (CDAI), have been developed to quantify various dimensions of diet quality, including overall adherence to dietary guidelines, inflammatory potential, and antioxidant capacity [8,9]. The HEI evaluates adherence to the Dietary Guidelines for Americans (DGA), while EDII estimates the pro-inflammatory effects of diet. CDAI, on the other hand, measures the antioxidant properties of dietary intake based on the consumption of vitamins, minerals, and other compounds known to reduce oxidative stress.

Despite the potential importance of these dietary indices, existing evidence on their associations with MASLD is inconsistent. Some studies have reported a protective role of high diet quality, as measured by CDAI, in reducing the risk of MASLD, while others have found no significant associations [9–11]. Similarly, past studies have explored the relationships between HEI, EDII, and MASLD, but many of these studies lacked comprehensive control for important covariates, such as physical activity (PA), poverty income ratio (PIR), and alcohol consumption [12,13]. These inconsistencies may be attributed to differences in methodologies, study populations, and the extent of covariate adjustments. Consequently, it is essential to revisit the relationship between dietary indices and MASLD using a robust methodological framework that accounts for a comprehensive set of confounding factors.

Beyond direct associations, the mechanisms underlying the relationship between dietary quality and MASLD remain inadequately explored. Insulin resistance, systemic inflammation, obesity or visceral fat distribution, and oxidative stress are well-established pathways in the pathogenesis of MASLD and may serve as mediators linking dietary quality to liver health [14–17]. For instance, diets rich in anti-inflammatory or antioxidant components may reduce insulin resistance and inflammation, thereby mitigating the risk of MASLD. However, few studies have systematically examined these pathways in the context of dietary indices and MASLD, leaving a significant gap in understanding.

This study aims to address these gaps by evaluating the associations between three dietary indices (HEI, EDII, and CDAI) and MASLD using data from the nationally representative National Health and Nutrition Examination Survey (NHANES) 2007–2018. We employed rigorous statistical models to control for a wide range of covariates, including sociodemographic factors, lifestyle behaviors, and metabolic health markers. Furthermore, we conducted a comprehensive mediation analysis to investigate the roles of potential mediators across four domains: insulin resistance (homeostatic model assessment of insulin resistance, HOMA-IR; metabolic score for insulin resistance, METS-IR), systemic inflammation (systemic inflammatory response index, SIRI; systemic immune-inflammation index, SII), obesity or visceral fat distribution (a body shape index, ABSI; body roundness index, BRI), and oxidative stress (Gamma-Glutamyltransferase, GGT; Bilirubin; Uric Acid). By examining these mediating pathways, this study seeks to provide novel insights into the mechanisms linking dietary quality to MASLD risk.

## 2. Methods

### 2.1 Study design and participants

This study utilizes data from the NHANES, a nationally representative, cross-sectional survey organized by the National Center for Health Statistics (NCHS). NHANES assesses the health and nutritional status of the noninstitutionalized population in the United States. The survey employs a precise stratified, multistage probability sampling design to ensure sample representativeness and reliability.

**Fig 1:**
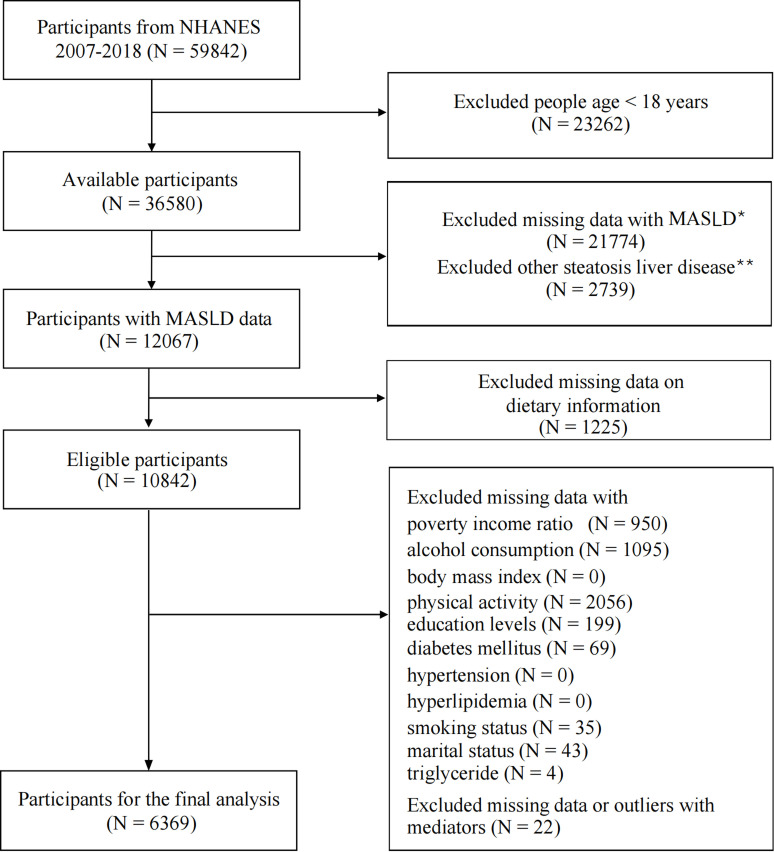
Flowchart of procedures for participants selection and inclusion. * “Excluded missing data with MASLD” refers to individuals with missing values for variables involved in the MASLD definitions and its related inclusion and exclusion criteria. **Other steatosis liver disease (SLD) refers to cryptogenic SLD, metabolic associated alcoholic liver disease (MetALD) or other combination aetiology SLD, and other specific aetiology SLD.

The present study uses data from the NHANES 2007–2018 cycles, including participants aged 18 years and older. The study was designed to evaluate the impact of the HEI on MASLD, while also investigating potential mediating variables. Participants with missing variables were excluded. A flowchart of participant inclusion and exclusion is shown in **Fig 1**. The final study sample consisted of 6,369 participants, with data representing a weighted population of 113,302,410 individuals.

The selection of the 2007–2018 cycles was made because these years provide the most comprehensive details on the United States Fatty Liver Index (USFLI) and Fatty Liver Index (FLI), which are crucial for the accurate diagnosis of MASLD. Additionally, the reason we did not combine the 2019–2020 cycles is due to the significant disruptions caused by the COVID-19 pandemic, which led to changes in the study’s methodology and sample collection protocols, making it unsuitable to combine these cycles with others. The FLI and USFLI have been proven reliable for diagnosing MASLD. Specifically, FLI has an area under the receiver operating characteristic curve of 0.78 (95% CI: 0.74–0.81), and the USFLI is 0.80 (95% CI: 0.77–0.83) [18,19]. Thus, based on these factors, the 2007–2018 cycles were selected for this study.

### 2.2 Ethics statement

This study was conducted under the auspices of the National Center for Health Statistics (NCHS), with comprehensive ethical oversight provided by the NCHS Institutional Review Board (IRB). Prior to data collection and health examinations, comprehensive informed consent was meticulously obtained from all eligible participants, ensuring full compliance with ethical research standards.

### 2.3 Definitions of dietary indices

For the dietary data, NHANES includes two 24-hour dietary recall interviews. In this study, we used the average of two 24-hour dietary recalls to obtain a more accurate representation of participants’ dietary intake

In this study, HEI specifically refers to the HEI-2015. It is designed to measure how closely an individual’s diet aligns with the DGA. A higher HEI score (ranging from 0 to 100) signifies better adherence to the DGA, indicating a more balanced and health-promoting dietary pattern.

The EDII is a dietary scoring system developed to assess the inflammatory potential of an individual’s diet. It is derived from a comprehensive review of the literature, analyzing over 1,900 peer-reviewed studies on the relationship between various dietary factors and inflammation. The score is adjusted for total energy intake, ensuring that the impact of diet-related inflammation is assessed independently of overall calorie consumption. An elevated EDII score reflects a diet that is more likely to promote inflammation.

The CDAI is an index used to quantify the antioxidant potential of an individual’s diet. It is based on the intake of key dietary antioxidants, including vitamins A, C, and E, carotenoids, as well as minerals such as zinc and selenium, which have been shown to have protective effects against oxidative stress and inflammation. A higher CDAI score indicates a diet with a higher antioxidant capacity.

For further details on the algorithms used to calculate HEI, EDII, and CDAI, please refer to previous studies [8,9].

### 2.4 Definitions of MASLD

Hepatic steatosis was identified using the USFLI or FLI. Specifically, hepatic steatosis was defined as an a USFLI score ≥  30 or FLI score ≥  60. The FLI and USFLI are both composite indices. For both indices, a higher score indicates a greater likelihood of hepatic steatosis. The calculation formulas for USFLI and FLI are as follows:


$$\text{USFLI}=\frac{e^{\left(\begin{array}{c} \text{0.3458*Mexican American-0.8073*non-Hispanic black+0.0093*age}\\ \text{+0.6151*InGGT+0.0249*waist circumference}\\ \text{+1.1792*Insulin+0.8242*InGlucose-14.7812} \end{array}\right)}}{1+e^{\left(\begin{array}{c} \text{0.3458*Mexican American-0.8073*non-Hispanic black+0.0093*age}\\ \text{+0.6151*InGGT+0.0249*waist circumference}\\ \text{+1.1792*Insulin+0.8242*InGlucose-14.7812} \end{array}\right)}}*100$$

$$\text{FLI}=\frac{{\text{e}}^{0.953\text{*In}\left(\text{TG}\right)+0.139\text{*BMI}+0.718\text{*In}\left(\text{GGT}\right)+0.053\text{*waist circumference}-15.745}}{1+{\text{e}}^{0.953\text{*In}\left(\text{TG}\right)+0.139\text{*BMI}+0.718\text{*In}\left(\text{GGT}\right)+0.053\text{*waist circumference}-15.745}}*100$$

The ethnicity factor assigns a value of 1 for participants identified as Mexican American or Non-Hispanic Black, and 0 for those not belonging to these groups.

MASLD was diagnosed in cases where hepatic steatosis was observed in the absence of the following conditions: (1) Alcohol consumption exceeding one drink per day for women or two drinks per day for men [15]; To define the status of alcohol consumption, we used the relevant “alq130” variable from the NHANES database. This variable specifically assesses the average number of alcoholic drinks consumed on days when participants reported drinking in the past 12 months. (2) Infection with Hepatitis B or C virus; (3) Use of pharmacological agents known to induce steatosis, including tamoxifen, amiodarone, nucleoside reverse transcriptase inhibitors, methotrexate, aspirin, ibuprofen, valproic acid, protease inhibitors, carbamazepine, fluorouracil, glucocorticoids and irinotecan [20]; (4) Iron overload, defined as a transferrin saturation of 45% or higher, combined with ferritin levels of at least 400 µg/L in women and 500 µg/L in men.

In accordance with the Delphi consensus definition, individuals with MASLD must have at least one of the five cardiometabolic risk factors. The specific cardiometabolic risk factors can be found in the referenced literature [12,21].

### 2.5 Definition of potential mediators

In this study, nine potential mediators were identified to represent four key biological aspects: IR (HOMA-IR, homeostatic model assessment of insulin resistance; METS-IR, metabolic score for insulin resistance), systemic inflammatory (SIRI, systemic inflammatory response index; SII, systemic immune-inflammation index), obesity or visceral fat distribution (ABSI, a body shape index; BRI, body roundness index), and oxidative stress (GGT, Gamma-Glutamyltransferase; Bilirubin; Uric Acid). Each mediator is described below:

HOMA-IR is a widely used index for assessing insulin resistance. It reflects the efficiency of insulin in regulating glucose homeostasis [22]. HOMA-IR was calculated using the formula FPG (mmol/L) × FINS (mIU/L)/22.5. METS-IR is a surrogate marker of insulin resistance. It has been validated as a reliable indicator of metabolic dysfunction associated with insulin resistance [22]. METS-IR =  Ln [2 × glycemia (mg/dL) +  triglycerides (mg/dL)] ×  BMI/Ln HDL-C (mg/dL).

SIRI and SII are both markers of systemic inflammation [23]. SIRI =  (neutrophil count ×  monocyte count)/ lymphocyte count, and SII =  (platelet count ×  neutrophil count)/ lymphocyte count.

BRI is a measure of body shape and visceral fat distribution.


$$\text{B}RI=364.2-365.5\times \sqrt{1-{\left(\frac{WC}{2\pi }\right)}^{2}/（0.5\times height{)}^{2}}$$

ABSI is another anthropometric index, offering a more nuanced evaluation of abdominal fat distribution and its associated health risks [24].


$$\text{ABSI}=\frac{\text{W}C\left(m\right)}{\text{B}MI{\left(\frac{kg}{m^{2}}\right)}^{\frac{2}{3}}\times height{\left(m\right)}^{\frac{1}{2}}}$$

GGT is an enzyme primarily involved in liver function and oxidative stress [25]. Bilirubin, a byproduct of hemoglobin breakdown, serves as an antioxidant and a marker of liver health. Altered bilirubin levels are linked to oxidative stress and metabolic disorders [25]. Uric acid, a byproduct of purine metabolism, is widely recognized as an indicator of metabolic health. Elevated levels of uric acid are associated with oxidative stress, systemic inflammation, and increased risk of metabolic syndrome [26].

Due to missing data in potential mediators and abnormalities in blood cell counts, the final number of participants included in the mediation analysis was 6,617. S1 Fig presents box plots illustrating the distribution of lymphocyte, monocyte, neutrophil, and platelet data, along with the identification and exclusion of outliers in blood cell count data to ensure the accuracy of the analysis.

### 2.6 Definition of covariates

These covariates include sociodemographic characteristics, physical measurements, lifestyle behaviors, prevalent health conditions and metabolic health markers, as outlined in Table 1. The methods used to collect and classify data on hyperlipidemia, hypertension, and diabetes mellitus (DM) are detailed in S1 Table. In the NHANES program, serum samples were obtained during laboratory assessments, including measurements of high-density lipoprotein (HDL) and triglycerides (TG), both of which are reported in units of mmol/L. Each participant completed a PA questionnaire covering activities performed in the past 30 days. The questionnaire recorded the type, frequency, and intensity of activities, categorized as moderate or vigorous [27,28]. Moderate activities involved slight increases in breathing or heart rate, while vigorous activities caused substantial increases. The weekly total PA volume (PA total MET) was calculated as the sum of MET scores from work, recreational, and transportation-related activities. Further details on the definition of covariates can be found in previously published studies [23].

**Table 1:** Descriptive characteristics of the study population stratified by MASLD.

Characteristic	Total	Non-MASLD	MASLD	*P*-values
	(N = 6369)	(N = 4338)	(N = 2031)	
**HEI**	54.33 ± 0.30	55.30 ± 0.36	52.11 ± 0.38	** < 0.001**
**EDII**	0.84 ± 0.03	0.79 ± 0.03	0.94 ± 0.05	**0.009**
**CDAI**	1.26 ± 0.09	1.41 ± 0.11	0.90 ± 0.12	** < 0.001**
**TG (mmol/L)**	1.29 ± 0.02	1.03 ± 0.01	1.89 ± 0.04	** < 0.001**
**HDL (mmol/L)**	1.45 ± 0.01	1.55 ± 0.01	1.20 ± 0.01	** < 0.001**
**PA total MET**	4848.7 ± 121.2	5040.5 ± 142.7	4410.9 ± 202.2	**0.008**
**METS-IR**	39.990 ± 0.195	34.484 ± 0.133	52.561 ± 0.323	** < 0.001**
**HOMA-IR**	2.937 ± 0.078	1.739 ± 0.024	5.675 ± 0.232	** < 0.001**
**SII**	493.93 ± 4.75	475.23 ± 5.33	536.64 ± 8.40	** < 0.001**
**SIRI**	1.11 ± 0.01	1.06 ± 0.01	1.25 ± 0.02	** < 0.001**
**BRI**	4.76 ± 0.04	3.83 ± 0.03	6.88 ± 0.07	** < 0.001**
**ABSI**	0.081 ± 0.001	0.080 ± 0.001	0.083 ± 0.001	** < 0.001**
**GGT**	22.72 ± 0.34	18.67 ± 0.31	31.98 ± 0.73	** < 0.001**
**Bilirubin**	0.72 ± 0.01	0.73 ± 0.01	0.70 ± 0.01	**0.004**
**Uric acid**	5.35 ± 0.02	5.07 ± 0.02	5.99 ± 0.04	** < 0.001**
**Age, n (%)**				** < 0.001**
18-29	1230 (21.47)	1056 (86.02)	174 (13.98)	
30-44	1638 (26.86)	1188 (73.43)	450 (26.57)	
45-59	1581 (27.94)	989 (63.63)	592 (36.37)	
>=60	1920 (23.73)	1105 (57.21)	815 (42.79)	
**Sex, n (%)**				** < 0.001**
Female	3102 (49.19)	2259 (75.96)	843 (24.04)	
Male	3267 (50.81)	2079 (63.34)	1188 (36.66)	
**Race/Ethnicity, n (%)**				** < 0.001**
Non‐Hispanic white	2969 (71.35)	2013 (68.91)	956 (31.09)	
Non‐Hispanic black	1208 (9.97)	835 (71.94)	373 (28.06)	
Hispanic	1383 (10.99)	856 (66.57)	527 (33.43)	
other race	809 (7.69)	634 (76.58)	175 (23.42)	
**BMI, n (%)**				** < 0.001**
Underweight/Normal	2350 (38.32)	2294 (98.14)	56 (1.86)	
Overweight	2275 (35.74)	1709 (76.30)	566 (23.70)	
Obese	1744 (25.94)	335 (17.99)	1409 (82.01)	
**Marital status, n (%)**				** < 0.001**
Married/Living with Partner	3924 (65.64)	2545 (66.64)	1379 (33.36)	
Never married	1248 (19.47)	1015 (81.29)	233 (18.71)	
Widowed/Divorced/Separated	1197 (14.89)	778 (66.98)	419 (33.02)	
**PIR, n (%)**				0.550
<1.3	1764 (18.17)	1178 (69.88)	586 (30.12)	
1.3-3.5	2322 (34.05)	1576 (68.45)	746 (31.55)	
>3.5	2283 (47.78)	1584 (70.20)	699 (29.80)	
**Education levels, n (%)**				** < 0.001**
Below high school	419 (3.30)	239 (61.58)	180 (38.42)	
High school	2076 (29.09)	1367 (66.14)	709 (33.86)	
Above high school	3874 (67.61)	2732 (71.40)	1142 (28.60)	
**Alcohol consumption, n (%)**				** < 0.001**
Never	877 (10.71)	523 (60.39)	354 (39.61)	
Former	1009 (12.77)	491 (47.43)	518 (52.57)	
Mild and above	4483 (76.52)	3324 (74.52)	1159 (25.48)	
**Smoking status, n (%)**				** < 0.001**
Never	3774 (59.48)	2569 (69.03)	1205 (30.97)	
Former	1503 (24.17)	915 (63.06)	588 (36.94)	
Now	1092 (16.35)	854 (80.99)	238 (19.01)	
**Hyperlipidemia, n (%)**				** < 0.001**
No	2099 (34.25)	1819 (87.82)	280 (12.18)	
Yes	4270 (65.75)	2519 (60.03)	1751 (39.97)	
**Hypertension, n (%)**				** < 0.001**
No	4044 (68.19)	3137 (78.66)	907 (21.34)	
Yes	2325 (31.81)	1201 (50.01)	1124 (49.99)	
**Diabetes mellitus, n (%)**				** < 0.001**
No	4330 (73.33)	3440 (79.27)	890 (20.73)	
Prediabetes	1018 (15.16)	540 (51.23)	478 (48.77)	
DM	1021 (11.51)	358 (31.70)	663 (68.30)	

**Abbreviations:** HEI, healthy eating index; EDII, energy density dietary inflammatory index; CDAI, composite dietary antioxidant index; TG, triglycerides; HDL, high density lipoprotein; PA, physical activity; MET, Metabolic Equivalent; BMI, body mass index; PIR, poverty income ratio. METS-IR, metabolic score for insulin resistance; HOMA-IR, homeostatic model assessment of insulin resistance; SII, systemic immune-inflammation index; SIRI, systemic inflammation response index; BRI, body roundness index; ABSI, a body shape index; GGT, serum gamma- glutamyltransferase. The normal reference values are as follows: TG: normal level < 1.7 mmol/L; HDL: men, normal level ≥ 1.0 mmol/L. women: normal level ≥ 1.3 mmol/L. *P*-value by chi-square test for classified variables.

### 2.7 Statistical analyses

For this study, we focused on data from the 2007–2018 survey cycles, combining six consecutive cycles. Dietary day one sample weight (WTSAF2YR*1/6) were applied to account for the combined survey cycles and ensure accurate population estimates. Descriptive statistics summarized participant characteristics, with continuous variables reported as means and standard deviations (SD) and categorical variables as frequencies and percentages. Differences between groups stratified by MASLD status were analyzed using Chi-square tests for categorical variables and ANOVA for continuous variables. Collinearity diagnostics were performed for all covariates, with variance inflation factors (VIF) calculated. All covariates had a VIF less than 2.3, indicating no multicollinearity. Statistical analyses were conducted using R Studio (version 4.3.1) with the nhanesR package (version 0.9.4.3), following the principles outlined in the STROBE Guidelines.

The HEI, EDII and CDAI were divided into quartiles, from the lowest group (Q1) to the highest group (Q4), as outlined in S2 Table. Weighted logistic regression was employed to assess the association between dietary indices and MASLD. Three regression models were developed: an unadjusted model with no covariate adjustments; Model 1 adjusted for sex, age, and race; and Model 2 further adjusted for BMI, PIR, education level, marital status, alcohol consumption, smoking status, hypertension, hyperlipidemia, DM, TG, HDL, and PA total MET. Associations between dietary indices and MASLD were assessed using odds ratios (OR) with corresponding 95% confidence intervals (CI). To ensure the assumptions of logistic regression were satisfied, we examined the linear relationship between continuous independent variables and the logit(p) transformation.

Subgroup analyses were performed to examine whether covariates potentially modified the relationship between dietary indices and MASLD. This approach enabled the association to be assessed within specific subgroups, such as age, sex, and BMI, ensuring the consistency of results across different population strata. Sensitivity analyses were conducted to address potential biases arising from missing data on key covariates. Notably, a substantial proportion of data was missing for PIR (N =  950), alcohol consumption (N =  1095) and PA total MET (N =  2056). A sensitivity analysis was conducted to test the robustness of the findings by excluding participants with missing data on PIR, alcohol consumption and PA total MET, resulting in a final sample of 10,083 participants.

**Fig 2:**
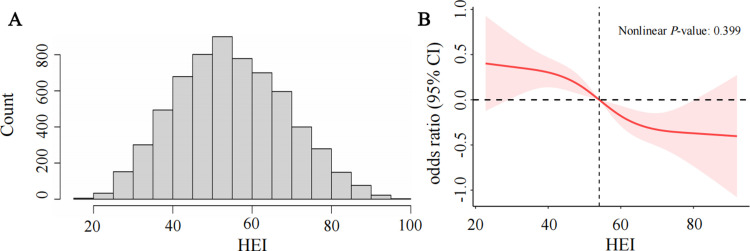
The distribution of HEI (A). The full-adjusted relationship between HEI and MASLD using Restricted Cubic Spline (B). The solid line represents the fitted nonlinearcurve. The area adjacent to the solid line represents the 95% confidence interval. Abbreviations: HEI, healthy eating index; CI, confidence interval.

We used histograms to assess the distribution of HEI (Fig 2A). Restricted cubic spline (RCS) analysis was utilized to examine potential nonlinear associations between dietary indices and MASLD. Nonlinear *P*-values were calculated to evaluate the significance.

**Fig 3:**
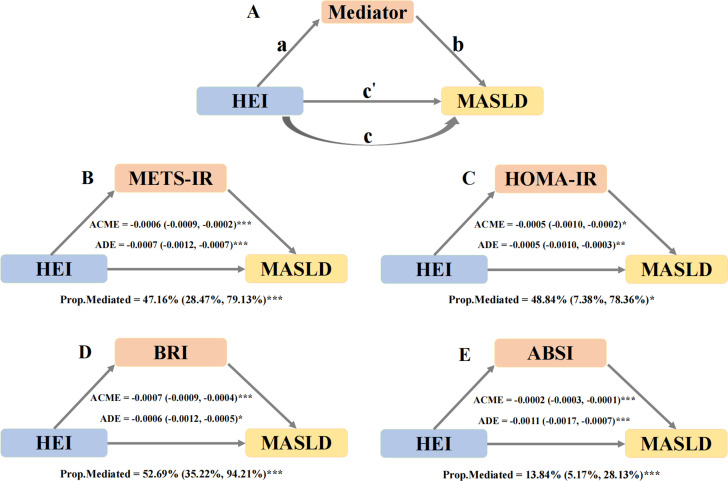
Mediation effects of potential mediators in the associations of HEI with MASLD. Notes: Adjust for age, sex, race, body mass index, poverty income ratio, education levels, marital status, smoking status, alcohol consumption, hyperlipidemia, hypertension, diabetes mellitus, triglyceride, high density lipoprotein and PA total MET. Abbreviations: HEI, healthy eating index; METS-IR, metabolic score for insulin resistance; HOMA-IR, homeostatic model assessment of insulin resistance; BRI, body roundness index; ABSI, a body shape index; ACME, average causal mediation effects (indirect effect); ADE, average direct effects. *  *P* <  0.05, ** *P* <  0.01, and *** *P* <  0.001.

The “Mediation” package was employed to perform mediation analysis to evaluate the mediating effects of potential mediators. The analysis followed a two-step approach, as shown in Fig 3A. First, regression models were applied to evaluate the influence of dietary indices on mediators (path a). Next, after adjusting for mediators, the effect of the mediators on MASLD (path b) and the effect of dietary indices on MASLD (path c’) were evaluated. The indirect effect was calculated as the product of path a and path b, and the mediation proportion was determined by dividing the indirect effect by the total effect. The total effect of dietary indices on MASLD was estimated without controlling for mediators (path c). Bootstrapping with 500 iterations was performed to calculate 95% confidence intervals for the mediation proportion.

## 3. Results

### 3.1 Descriptive characteristics

A total of 6,369 participants were included in this study (Fig 1). Table 1 summarizes the characteristics of the study population, stratified by MASLD status. The prevalence of MASLD increased significantly with age (*P* <  0.001), with the highest prevalence observed in participants aged ≥ 60 years (42.79%). Among the participants, 3,102 (49.19%) were female, and 3,267 (50.81%) were male. The prevalence of MASLD was significantly higher in males compared to females (*P* <  0.001). Regarding BMI categories, 2,350 (38.32%) participants were under weight or normal weight, 2,275 (35.74%) were overweight, and 1,744 (25.94%) were obese. The prevalence of MASLD increased significantly with BMI, with 82.01% of obese participants having MASLD (*P* <  0.001). Other race had the lowest prevalence of MASLD (23.42%), while Hispanics had the highest prevalence (33.43%). Notable variations in MASLD prevalence were identified among racial/ethnic groups (*P* <  0.001). Participants with a PIR 1.3–3.5 had the highest prevalence of MASLD (31.55%), although the differences among PIR categories were not significant (*P* =  0.550). The prevalence of MASLD was highest among participants who had not completed high school (38.42%, *P* <  0.001). Other factors significantly associated with higher MASLD prevalence included being married/living with partner, being a former smoker, having hyperlipidemia, hypertension, or DM (*P* <  0.001 for all).

MASLD participants had significantly lower HEI scores (52.11 ±  0.38 vs 55.30 ±  0.36, *P* <  0.001) and CDAI scores (0.90 ±  0.12 vs 1.41 ±  0.11, *P* <  0.001) compared to non-MASLD participants, whereas EDII had significantly higher scores (0.94 ±  0.05 vs 0.79 ±  0.03, *P* =  0.009). Additionally, participants with MASLD had significantly higher TG levels and lower HDL and PA total MET values compared to those without MASLD (*P* <  0.01 for all).

### 3.2 Binary logistic regression analysis

Binary logistic regression was utilized to examine the associations between dietary indices and MASLD, with results detailed in **Table 2**. In the unadjusted model, HEI (continuous) demonstrated a significant inverse association with MASLD (OR: 0.98, 95% CI: 0.98–0.99, *P* <  0.001). This association persisted after adjusting for sex, age, and race in Model 1 (OR: 0.97, 95% CI: 0.97–0.98, *P* <  0.001) and remained significant in Model 2, which included all covariates (OR: 0.98, 95% CI: 0.97–0.99, *P* <  0.001).

**Table 2:** Adjusted association of dietary indexs with MASLD.

Exposure	Unadjusted model	Adjust 1	Adjust 2
	Odds ratio (95% CI) associated with MASLD		
**HEI (continuous)**	0.98 (0.98, 0.99); **< 0.001**	0.97 (0.97, 0.98); **< 0.001**	0.98 (0.97, 0.99); **< 0.001**
**Quartile of HEI**			
Q1	1 (Ref)	1 (Ref)	1 (Ref)
Q2	0.92 (0.78, 1.08); 0.300	0.82 (0.69, 0.98); **0.030**	0.83 (0.61, 1.12); 0.210
Q3	0.71 (0.60, 0.84); < **0.001**	0.58 (0.49, 0.70); **< 0.001**	0.66 (0.49, 0.89); **0.010**
Q4	0.53 (0.44, 0.63); **< 0.001**	0.40 (0.33, 0.48); **< 0.001**	0.46 (0.33, 0.64); **< 0.001**
*P* for trend	** < 0.001**	** < 0.001**	** < 0.001**
**EDII** **(continuous)**	1.05 (1.01, 1.10); **0.020**	1.12 (1.06, 1.18); **0.001**	1.03 (0.97, 1.10); 0.332
**Quartile of EDII**			
Q1	1 (Ref)	1 (Ref)	1 (Ref)
Q2	1.40 (1.14, 1.73); **0.002**	1.58 (1.29, 1.94); **< 0.001**	1.16 (0.83, 1.62); 0.380
Q3	1.43 (1.17, 1.76); **< 0.001**	1.80 (1.45, 2.23); **< 0.001**	1.14 (0.80, 1.63); 0.472
Q4	1.41 (1.13, 1.77); **0.003**	1.98 (1.60, 2.46); **< 0.001**	1.20 (0.85, 1.71); 0.301
*P* for trend	**0.002**	** < 0.001**	0.320
**CDAI (continuous)**	0.97 (0.96, 0.99); **0.001**	0.96 (0.95, 0.98); **< 0.001**	0.99 (0.97, 1.02); 0.663
**Quartile of CDAI**			
Q1	1 (Ref)	1 (Ref)	1 (Ref)
Q2	0.94 (0.78, 1.13); 0.513	0.87 (0.72, 1.04); 0.136	0.80 (0.57, 1.12); 0.192
Q3	0.94 (0.76, 1.16); 0.542	0.86 (0.69, 1.07); 0.163	0.87 (0.65, 1.18); 0.372
Q4	0.77 (0.62, 0.96); **0.020**	0.70 (0.57, 0.87); **0.001**	0.91 (0.62, 1.32); 0.613
*P* for trend	**0.022**	**0.001**	0.367

Unadjusted model: non-adjusted model.

Adjust 1: Adjust for age, sex, race.

Adjust 2: Adjust for age, sex, race, body mass index, poverty income ratio, education levels, marital status, smoking status, alcohol consumption, hyperlipidemia, hypertension, diabetes mellitus, triglyceride, high density lipoprotein and PA total MET. **Abbreviations**: HEI, healthy eating index; EDII, energy density dietary inflammatory index; CDAI, composite dietary antioxidant index; CI, confidence interval.

When HEI was analyzed as quartiles (Q1–Q4), consistent and significant associations were observed after full adjustment (Model 2). Participants in the highest quartile (Q4) exhibited substantially lower odds of MASLD compared to those in the lowest quartile (Q1) (OR: 0.46, 95% CI: 0.33–0.64, *P* <  0.001). The OR for Q2 and Q3 were 0.83 (95% CI: 0.61–1.12, *P* =  0.210) and 0.66 (95% CI: 0.49–0.89, *P* =  0.010), respectively. The *P* for trend in all models was also highly significant (*P* <  0.001).

In contrast, no significant associations were identified between MASLD and either EDII or CDAI after adjusting for all covariates in Model 2. This indicates that HEI was the only dietary index consistently and significantly associated with MASLD following comprehensive adjustment. Given the consistent and significant association between HEI and MASLD across all models, HEI was selected for subsequent analyses.

### 3.3 Subgroup analyses and sensitivity analysis

The results of the subgroup analysis are shown in **Table 3**. After controlling for all covariates, no significant interactions were observed in subgroups stratified by sex, age, BMI, race, smoking status, hyperlipidemia, hypertension, and DM, as all *P*-values for interaction exceeded 0.05. This suggests that the association between HEI and MASLD was consistent across these groups. To assess the robustness of these results, sensitivity analyses were performed (S3 Table). The outcomes were aligned with those in **Table 2**, confirming that HEI remained significantly associated with MASLD across all models.

**Table 3:** Adjusted association of HEI with MASLD for subgroup analyses.

Subgroups	Q2	Q3	Q4	*P* for interaction
Adjusted odds ratio (95% CI); *P* ^*^			
**Sex**				0.998
Female	0.90 (0.56, 1.45); 0.658	0.72 (0.43, 1.20); 0.202	0.50 (0.30, 0.83); **0.008**	
Male	0.76 (0.47, 1.24); 0.268	0.61 (0.39, 0.95); **0.028**	0.42 (0.25, 0.71); **0.001**	
**Age**				0.263
18-29	1.21 (0.53, 2.76); 0.642	0.67 (0.28, 1.62); 0.373	0.49 (0.16, 1.54); 0.218	
30-44	0.83 (0.43, 1.60); 0.573	0.98 (0.49, 1.94); 0.732	0.58 (0.26, 1.30); 0.182	
45-59	0.63 (0.35, 1.15); 0.134	0.45 (0.25, 0.81); **0.009**	0.25 (0.12, 0.52); **< 0.001**	
>=60	1.02 (0.55, 1.87); 0.955	0.76 (0.41, 1.38); 0.360	0.66 (0.37, 1.17); 0.154	
**BMI**				0.101
Underweight/Normal	0.24 (0.07, 0.73); **0.024**	0.58 (0.18, 1.72); 0.300	0.17 (0.04, 0.68); **0.013**	
Overweight	1.10 (0.71, 1.72); 0.660	0.63 (0.39, 1.02); 0.060	0.68 (0.43, 1.05); 0.083	
Obese	0.61 (0.32, 1.16); 0.129	0.73 (0.40, 1.34); 0.306	0.28 (0.14, 0.54); **< 0.001**	
**Race/Ethnicity**				0.189
Non‐Hispanic white	0.76 (0.50, 1.16); 0.206	0.71 (0.47, 1.07); 0.101	0.39 (0.25, 0.61); **< 0.001**	
Non‐Hispanic black	0.91 (0.47, 1.71); 0.767	0.49 (0.27, 0.90); **0.023**	0.43 (0.19, 0.91); **0.030**	
Hispanic	0.85 (0.45, 1.61); 0.610	0.66 (0.34, 1.31); 0.233	0.82 (0.44, 1.54); 0.533	
other race	1.88 (0.49, 7.30); 0.351	0.58 (0.15, 2.21); 0.417	0.60 (0.16, 2.33); 0.457	
**Smoking status**				0.104
Never	0.90 (0.57, 1.41); 0.629	0.53 (0.34, 0.83); **0.006**	0.38 (0.25, 0.60); **< 0.001**	
Former	0.59 (0.35, 1.02); 0.057	0.73 (0.41, 1.31); 0.293	0.51 (0.26, 1.01); **0.050**	
Now	0.74 (0.34, 1.57); 0.42	0.74 (0.30, 1.88); 0.526	0.85 (0.30, 2.37); 0.748	
**Hyperlipidemia**				0.709
No	0.80 (0.33, 1.96); 0.628	0.89 (0.51, 1.54); 0.666	0.63 (0.25, 1.45); 0.253	
Yes	0.82 (0.56, 1.20); 0.309	0.63 (0.43, 0.91); **0.016**	0.44 (0.30, 0.63); **< 0.001**	
**Hypertension**				0.815
No	0.94 (0.58, 1.54); 0.813	0.74 (0.46, 1.17); 0.190	0.55 (0.34, 0.91); **0.020**	
Yes	0.75 (0.45, 1.24); 0.251	0.59 (0.36, 0.98); **0.041**	0.37 (0.21, 0.64); **< 0.001**	
**Diabetes mellitus**				0.839
No	0.88 (0.58, 1.35); 0.568	0.73 (0.49, 1.08); 0.112	0.46 (0.29, 0.73); **0.001**	
Prediabetes	0.52 (0.25, 1.01); 0.079	0.65 (0.30, 1.44); 0.286	0.40 (0.19, 0.86); **0.020**	
DM	0.71 (0.28, 1.84); 0.481	0.48 (0.20, 1.12); 0.089	0.49 (0.19, 1.27); 0.141	

* We used the lowest quartile as the reference category. Outcome variable: MASLD. Fully adjusted model: adjust for age, sex, race, body mass index, poverty income ratio, education levels, marital status, smoking status, alcohol consumption, hyperlipidemia, hypertension, diabetes mellitus, triglyceride, high density lipoprotein and PA total MET, but not for the specific stratification variables of interest.

**Abbreviations:** BMI, body mass index; HEI, healthy eating index; CI, confidence interval.

In summary, the subgroup and sensitivity analyses consistently demonstrated that HEI was significantly associated with MASLD across different population strata and analytical approaches, confirming the reliability and robustness of the primary results.

### 3.4 Nonlinear relationships explore

To explore the potential nonlinear association between HEI and MASLD, we applied an RCS model with 4 strategically positioned knots, as illustrated in Fig 2B. After adjusting for all covariates, the analysis indicated no statistically significant nonlinear relationship between HEI and MASLD risk (nonlinearity *P* =  0.399). This result aligns with the statistically significant *P* for trend observed in Table 2, suggesting a linear association.

As shown in S4 Table, we conducted sensitivity analyses by varying the number of knots from 3 to 8. Regardless of the number of knots used in the RCS model, the nonlinearity *P*-values were consistently greater than 0.05, indicating no evidence of a nonlinear relationship between HEI and MASLD across different knot selections. These findings further support the robustness of the observed linear relationship.

### 3.5 Mediation analysis

In the mediation analysis, HEI, potential mediators, and MASLD were treated as the independent variable, mediator variables, and dependent variable, respectively. The relationship between HEI (continuous) and potential mediators was explored. As shown in S5 Table, in Model 2, METS-IR, HOMA-IR, SII, SIRI, ABSI, BRI, and bilirubin were significantly associated with HEI, indicating that the path a was significant for these mediators.

Next, the relationship between potential mediators and MASLD was examined. As shown in S6 Table, after controlling all covariates and HEI, METS-IR, HOMA-IR, BRI, ABSI, and GGT were significantly associated with MASLD in Model 2, indicating that the path b was significant for these mediators.

To satisfy the prerequisites for mediation analysis, mediators must exhibit significant associations in both the paths a and b. Among the potential mediators, METS-IR, HOMA-IR, BRI, and ABSI met this criterion. These four mediators were selected for mediation analysis (Fig 3). The mediation analysis revealed significant indirect effects of HEI on MASLD through all four mediators. For METS-IR, the mediation proportion was 47.16% (95% CI: 28.47%–79.13%, *P* <  0.001). For HOMA-IR, the mediation proportion was 48.84% (95% CI: 7.38%–78.36%, *P* <  0.05). For BRI, the mediation proportion was 52.69% (95% CI: 35.22%–94.21%, *P* <  0.001). For ABSI, the mediation proportion was 13.84% (95% CI: 5.17%–28.13%, *P* <  0.001). These findings indicate that a portion of the relationship between HEI and MASLD is mediated by these factors, as shown in Fig 3.

## 4. Discussion

This study highlights the association between dietary quality, reflected by the HEI, and MASLD risk. HEI demonstrated a significant inverse association with MASLD, whereas the EDII and CDAI showed no significant associations. Mediation analysis revealed that insulin resistance (METS-IR, HOMA-IR) and visceral fat distribution (BRI, ABSI) partially mediated the relationship between HEI and MASLD, underscoring the importance of metabolic pathways in linking diet to liver health.

Our analysis identified distinct patterns in MASLD prevalence across socio-demographic groups. Younger individuals, females, those with underweight/normal weight, and greater educational attainment were found to have lower MASLD prevalence. These results are broadly consistent with prior studies analyzing national datasets, which have underscored the influence of social factors on liver health [12,29]. These findings emphasize the importance of considering these covariates when studying the relationship between HEI and MASLD. Moreover, our findings show that MASLD is more prevalent in individuals with hyperlipidemia, hypertension or DM, conditions that are strongly implicated in metabolic dysfunction [30–32]. Hyperlipidemia is strongly associated with MASLD, with studies showing that moderate and severe hyperlipidemia significantly increase the prevalence of MASLD [33]. Hypertension is recognized as an independent risk factor for MASLD, with studies indicating that early-stage hypertension may promote MASLD development even without other metabolic abnormalities [34]. Additionally, managing blood pressure may help in preventing or slowing the progression of MASLD [34]. Similarly, DM contributes to insulin resistance and lipid dysregulation, creating conditions favorable for MASLD development [35]. Previous studies have highlighted these connections, and our findings support these established relationships while reinforcing the need for integrated metabolic risk management in MASLD prevention.

The relationship between iron overload and MASLD is complex. Recent studies have indicated that elevated serum ferritin levels are closely associated with the occurrence and severity of MASLD. However, iron overload is not merely a characteristic of MASLD; it may also reflect systemic inflammation or other metabolic abnormalities [36]. Research suggests that iron overload, particularly hyperferritinemia, may contribute to hepatic fat deposition and exacerbate liver fibrosis [37]. Nonetheless, some MASLD patients may present with concomitant hereditary hemochromatosis or other secondary causes of iron overload, which could independently impact liver health rather than being a direct component of MASLD [38,39]. To ensure the homogeneity of our study population and exclude potential liver diseases primarily driven by iron metabolism disorders, we excluded individuals with significant iron overload.

Prior studies examining dietary indices and MASLD have reported conflicting results, likely due to differences in population characteristics, study design, and the extent of covariate adjustments. While some studies have highlighted the protective effects of CDAI on MASLD, others found no association [9–11]. Our findings strengthen the evidence supporting CDAI not a key dietary index associated with MASLD risk, leveraging a large, nationally representative dataset and rigorous analytical methods. Similarly, EDII did not exhibit significant associations with MASLD in this study. While these indices capture specific dietary components, such as antioxidant properties and inflammatory potential, they may not fully reflect the broader dietary patterns encompassed by HEI. This suggests that overall dietary quality, rather than individual dietary properties, may play a more critical role in MASLD prevention.

A novel contribution of this study is the identification of insulin resistance and visceral fat distribution as mediators in the HEI-MASLD relationship. Insulin resistance is a hallmark of MASLD pathogenesis, promoting hepatic lipid accumulation and impairing metabolic regulation [40]. HEI, characterized by higher consumption of nutrient-dense foods, may enhance insulin sensitivity and reduce insulin resistance, thereby mitigating MASLD risk [14]. Similarly, visceral fat distribution, reflected by BRI and ABSI, is strongly linked to MASLD [41]. Visceral adiposity contributes to systemic inflammation, and metabolic syndrome, both of which exacerbate MASLD progression [41,42]. The inverse association between diet quality and visceral fat suggests that HEI may influence MASLD through visceral fat pathway [43]. These findings expand the understanding of how dietary patterns influence liver health and highlight the need for interventions targeting these mediating pathways.

Oxidative stress plays a critical role in the pathogenesis and progression of MASLD [44]. Diet has been widely recognized as a key factor influencing oxidative stress levels, with dietary patterns either exacerbating or mitigating oxidative damage [45]. Given the well-established link between oxidative stress and MASLD, we included oxidative stress as one of the potential mediators in our analysis to explore whether it contributes to the association between dietary indices and MASLD risk. To assess oxidative stress, we selected GGT, Bilirubin, and Uric Acid as biomarkers, as they are commonly used indicators of systemic oxidative stress in epidemiological and clinical studies [46]. By incorporating these oxidative stress markers into our mediation analysis, we aimed to determine whether oxidative stress acts as a biological link between dietary quality and MASLD risk.

The results of this study carry important implications for the prevention and management of MASLD. First, promoting adherence to dietary guidelines, as reflected by higher HEI scores, represents a practical and effective strategy for reducing MASLD prevalence. Public health campaigns emphasizing the benefits of high-quality diets rich in vegetables, fruits, lean proteins, and whole grains could play a pivotal role in addressing the growing burden of MASLD. The relationship between dietary composition and MASLD is well documented. A Western-style dietary pattern, characterized by high intake of red and processed meats, refined sugars, and saturated fats, along with low consumption of fiber-rich foods, has been associated with an increased risk of hepatic steatosis and liver fibrosis [47]. Conversely, adherence to healthier dietary patterns, such as the Mediterranean diet, has been shown to reduce liver fat accumulation, improve insulin sensitivity, and lower inflammation, thereby exerting protective effects against MASLD [48]. The observed lower HEI scores in MASLD patients in our study, reinforcing the need for dietary interventions targeting specific nutrient imbalances rather than focusing solely on total caloric intake. Additionally, integrating dietary assessments into clinical practice may help identify at-risk individuals and guide personalized nutritional interventions. Second, targeting mediating factors such as insulin resistance and visceral fat distribution may enhance the effectiveness of MASLD prevention efforts. Interventions aimed at improving metabolic health through diet and lifestyle modifications could reduce the burden of MASLD. These findings also underscore the need for multidisciplinary approaches combining dietary counseling, metabolic management, and lifestyle interventions in MASLD care.

This study has several methodological strengths that enhance the reliability and generalizability of its findings. The use of a large, nationally representative sample from NHANES (2007–2018) ensures that the results are applicable to diverse populations in the United States. The rigorous analytical framework, including comprehensive covariate adjustments and mediation analysis, provides robust insights into the complex relationships between dietary quality, metabolic mediators, and MASLD. Additionally, the inclusion of multiple dietary indices and mediators spanning four domains—insulin resistance, systemic inflammation, visceral fat distribution, and oxidative stress—offers a comprehensive evaluation of the pathways linking diet to MASLD.

Despite its strengths, several limitations should be considered when interpreting these findings. The cross-sectional design of NHANES data limits the ability to draw causal conclusions, and longitudinal studies are needed to confirm the observed associations and mediation effects. Furthermore, dietary intake was evaluated using self-reported 24-hour recalls, which are prone to recall bias and potential underreporting. While HEI demonstrated strong associations with MASLD, the lack of significant findings for EDII and CDAI warrants further investigation. Future studies should explore whether these indices may have stronger associations with specific subgroups or stages of MASLD. Another limitation is the reliance on surrogate markers, such as FLI and USFLI, to define MASLD. While these indices are validated and widely used, direct imaging or biopsy-based measures of liver fat would provide more definitive assessments. Moreover, the mediators examined in this study do not capture all potential pathways, such as gut microbiota alterations, which may also play critical roles in the diet-MASLD relationship.

## 5. Conclusion

In conclusion, this study establishes HEI as a key dietary factor associated with reduced MASLD risk, with insulin resistance and visceral fat distribution playing mediating roles. These findings provide actionable insights into the mechanisms linking diet to liver health and underscore the importance of promoting high-quality diets in MASLD prevention.

## Supporting information

S1 Fig Box plots showing the distribution of data for Lymphocyte (A), Monocyte (B), Neutrophil (C), and Platelet (D). Outliers are highlighted within the black rectangular boxes.
(TIF)

S1 Table The detailed overview of how we obtained information on hypertension, hyperlipidemia, and diabetes. (DOCX)

S2 Table Details of dietary indexs division. (DOCX)

S3 Table Adjusted association of dietary indexs with MASLD for sensitivity analysis. Unadjusted model: non-adjusted model. Adjust 1: Adjust for age, sex, race. Adjust 2: Adjust for age, sex, race, body mass index, education levels, marital status, e density lipoprotein. Abbreviations: HEI, healthy eating index; CI, confidence interval.
(DOCX)

S4 Table Nonlinear *P*-values of HEI and MASLD at different knots. Abbreviations: HEI, healthy eating index; CI, confidence interval.
(DOCX)

S5 Table Relationship between HEI and potential mediators in different models. Unadjusted model: non-adjusted model. Adjust 1: Adjust for age, sex, race. Adjust 2: Adjust for age, sex, race, body mass index, poverty income ratio, education levels, marital status, smoking status, alcohol consumption, hyperlipidemia, hypertension, diabetes mellitus, triglyceride, high density lipoprotein and PA total MET. Abbreviations: HEI, healthy eating index; METS-IR, metabolic score for insulin resistance; HOMA-IR, homeostatic model assessment of insulin resistance; SII, systemic immune-inflammation index; SIRI, systemic inflammation response index; BRI, body roundness index; ABSI, a body shape index; GGT, serum gamma- glutamyltransferase; CI, confidence interval.
(DOCX)

S6 Table Relationship between HEI and potential mediators with MASLD in different models. Unadjusted model: non-adjusted model. Adjust 1: Adjust for age, sex, race. Adjust 2: Adjust for age, sex, race, body mass index, poverty income ratio, education levels, marital status, smoking status, alcohol consumption, hyperlipidemia, hypertension, diabetes mellitus, triglyceride, high density lipoprotein and PA total MET. Abbreviations: HEI, healthy eating index; METS-IR, metabolic score for insulin resistance; HOMA-IR, homeostatic model assessment of insulin resistance; SII, systemic immune-inflammation index; SIRI, systemic inflammation response index; BRI, body roundness index; ABSI, a body shape index; GGT, serum gamma- glutamyltransferase; CI, confidence interval. * To address the extreme OR values observed during the initial analysis, we scaled the ABSI values by multiplying them by 100. This transformation ensured that the variable was within a more interpretable and computationally stable range, without affecting the underlying associations. After this adjustment, the logistic regression model yielded reasonable and reliable OR estimates.
(DOCX)

## References

[1] Lazarus JV, Newsome PN, Francque SM, Kanwal F, Terrault NA, Rinella ME. Reply: A multi-society Delphi consensus statement on new fatty liver disease nomenclature. Hepatology. 2024;79(3):E93–4. doi: 10.1097/HEP.0000000000000696 37983810

[2] Huttasch M, Roden M, Kahl S. Obesity and MASLD: Is weight loss the (only) key to treat metabolic liver disease? Metabolism. 2024;157:155937. doi: 10.1016/j.metabol.2024.155937 38782182

[3] Stefan N, Häring H-U, Cusi K. Non-alcoholic fatty liver disease: causes, diagnosis, cardiometabolic consequences, and treatment strategies. Lancet Diabetes Endocrinol. 2019;7(4):313–24. doi: 10.1016/S2213-8587(18)30154-2 30174213

[4] Driessen S, Francque SM, Anker SD, Castro Cabezas M, Grobbee DE, Tushuizen ME, et al. Metabolic dysfunction-associated steatotic liver disease and the heart. Hepatology. 2023:10.1097/HEP.0000000000000735. doi: 10.1097/HEP.0000000000000735 38147315 PMC12266800

[5] Zhang H, Zhou X-D, Shapiro MD, Lip GYH, Tilg H, Valenti L, et al. Global burden of metabolic diseases, 1990-2021. Metabolism. 2024;160:155999. doi: 10.1016/j.metabol.2024.155999 39151887

[6] Zelber-Sagi S, Carrieri P, Pericàs JM, Ivancovsky-Wajcman D, Younossi ZM, Lazarus JV. Food inequity and insecurity and MASLD: burden, challenges, and interventions. Nat Rev Gastroenterol Hepatol. 2024;21(10):668–86. doi: 10.1038/s41575-024-00959-4 39075288

[7] Zeng X-F, Varady KA, Wang X-D, Targher G, Byrne CD, Tayyem R, et al. The role of dietary modification in the prevention and management of metabolic dysfunction-associated fatty liver disease: An international multidisciplinary expert consensus. Metabolism. 2024;161:156028. doi: 10.1016/j.metabol.2024.156028 39270816

[8] Jayanama K, Theou O, Godin J, Cahill L, Shivappa N, Hébert JR, et al. Relationship between diet quality scores and the risk of frailty and mortality in adults across a wide age spectrum. BMC Med. 2021;19(1):64. doi: 10.1186/s12916-021-01918-5 33722232 PMC7962372

[9] Yang Z, Song S, Li L, Yuan Z, Li Y. Association between the composite dietary antioxidant index and metabolic dysfunction-associated steatotic liver disease in adults: a cross-sectional study from NHANES 2017-2020. Sci Rep. 2024;14(1):13801. doi: 10.1038/s41598-024-63965-1 38877074 PMC11178812

[10] Zhang Z, Wang H, Chen Y. Association between composite dietary antioxidant index and metabolic dysfunction associated steatotic liver disease: result from NHANES, 2017-2020. Front Nutr. 2024;11:1412516. doi: 10.3389/fnut.2024.1412516 39104752 PMC11299214

[11] He Y, Ye M, Xia Y, Zhong Z, Li Q. Antioxidants and the risk of metabolic dysfunction-associated steatotic liver disease: results of National Health and Nutrition Examination Survey and two-sample Mendelian randomization analyses. Eur J Gastroenterol Hepatol. 2025;37(2):230–9. doi: 10.1097/MEG.0000000000002898 39621882

[12] Xu M, Zhan Y, Gao G, Zhu L, Wu T, Xin G. Associations of five dietary indices with metabolic dysfunction-associated steatotic liver disease and liver fibrosis among the United States population. Front Nutr. 2024;11:1446694. doi: 10.3389/fnut.2024.1446694 39221157 PMC11363712

[13] Doustmohammadian A, Zamani F, Hébert JR, Moradi-Lakeh M, Esfandyiari S, Amirkalali B, et al. Exploring the link between dietary inflammatory index and NAFLD through a structural equation modeling approach. J Health Popul Nutr. 2024;43(1):224. doi: 10.1186/s41043-024-00721-1 39719637 PMC11668019

[14] Mambrini SP, Grillo A, Colosimo S, Zarpellon F, Pozzi G, Furlan D, et al. Diet and physical exercise as key players to tackle MASLD through improvement of insulin resistance and metabolic flexibility. Front Nutr. 2024;11:1426551. doi: 10.3389/fnut.2024.1426551 39229589 PMC11370663

[15] Wang Y, Chen S, Tian C, Wang Q, Yang Z, Che W, et al. Association of systemic immune biomarkers with metabolic dysfunction-associated steatotic liver disease: a cross-sectional study of NHANES 2007-2018. Front Nutr. 2024;11:1415484. doi: 10.3389/fnut.2024.1415484 39296508 PMC11408230

[16] Mai Z, Chen Y, Mao H, Wang L. Association between the skeletal muscle mass to visceral fat area ratio and metabolic dysfunction-associated fatty liver disease: A cross-sectional study of NHANES 2017-2018. J Diabetes. 2024;16(6):e13569. doi: 10.1111/1753-0407.13569 38751375 PMC11096813

[17] Gensluckner S, Wernly B, Datz C, Aigner E. Iron, Oxidative Stress, and Metabolic Dysfunction-Associated Steatotic Liver Disease. Antioxidants (Basel). 2024;13(2):208. doi: 10.3390/antiox13020208 38397806 PMC10886327

[18] Ruhl CE, Everhart JE. Fatty liver indices in the multiethnic United States National Health and Nutrition Examination Survey. Aliment Pharmacol Ther. 2015;41(1):65–76. doi: 10.1111/apt.13012 25376360

[19] Bedogni G, Bellentani S, Miglioli L, Masutti F, Passalacqua M, Castiglione A, et al. The Fatty Liver Index: a simple and accurate predictor of hepatic steatosis in the general population. BMC Gastroenterol. 2006;6:33. doi: 10.1186/1471-230X-6-33 17081293 PMC1636651

[20] Li L, Huang Q, Yang L, Zhang R, Gao L, Han X, et al. The Association between Non-Alcoholic Fatty Liver Disease (NAFLD) and Advanced Fibrosis with Serological Vitamin B12 Markers: Results from the NHANES 1999-2004. Nutrients. 2022;14(6):1224. doi: 10.3390/nu14061224 35334881 PMC8948655

[21] Rinella ME, Lazarus JV, Ratziu V, Francque SM, Sanyal AJ, Kanwal F, et al. A multisociety Delphi consensus statement on new fatty liver disease nomenclature. Hepatology. 2023;78(6):1966–86. doi: 10.1097/HEP.0000000000000520 37363821 PMC10653297

[22] Duan M, Zhao X, Li S, Miao G, Bai L, Zhang Q, et al. Metabolic score for insulin resistance (METS-IR) predicts all-cause and cardiovascular mortality in the general population: evidence from NHANES 2001-2018. Cardiovasc Diabetol. 2024;23(1):243. doi: 10.1186/s12933-024-02334-8 38987779 PMC11238348

[23] Ren Z, Xue Y, Zhang H, Guo T, Yi W, Li L, et al. Systemic Immune-Inflammation Index and Systemic Inflammation Response Index are Associated With Periodontitis: Evidence From NHANES 2009 to 2014. Int Dent J. 2024;74(5):1033–43. doi: 10.1016/j.identj.2024.03.019 38688802 PMC11561492

[24] Chen Y, Ding Y, Jin S, Zhang Y. Association between a body shape index and cognitive impairment among US older adults aged 40 years and above from a cross-sectional survey of the NHANES 2011-2014. Front Endocrinol (Lausanne). 2024;15:1411701. doi: 10.3389/fendo.2024.1411701 39377074 PMC11456444

[25] Whitfield JB. Serum gamma-glutamyltransferase and risk of disease. Clin Chem. 2007;53(1):1–2. doi: 10.1373/clinchem.2006.080911 17202494

[26] Gherghina M-E, Peride I, Tiglis M, Neagu TP, Niculae A, Checherita IA. Uric Acid and Oxidative Stress-Relationship with Cardiovascular, Metabolic, and Renal Impairment. Int J Mol Sci. 2022;23(6):3188. doi: 10.3390/ijms23063188 35328614 PMC8949471

[27] Liu C, Hua L, Xin Z. Synergistic impact of 25-hydroxyvitamin D concentrations and physical activity on delaying aging. Redox Biol. 2024;73:103188. doi: 10.1016/j.redox.2024.103188 38740004 PMC11103937

[28] Ainsworth BE, Haskell WL, Whitt MC, Irwin ML, Swartz AM, Strath SJ, et al. Compendium of physical activities: an update of activity codes and MET intensities. Med Sci Sports Exerc. 2000;32(9 Suppl):S498-504. doi: 10.1097/00005768-200009001-00009 10993420

[29] Wu Y, Tan Z, Zhen J, Liu C, Zhang J, Liao F, et al. Association between diet soft drink consumption and metabolic dysfunction-associated steatotic liver disease: findings from the NHANES. BMC Public Health. 2023;23(1):2286. doi: 10.1186/s12889-023-17223-0 37985986 PMC10658943

[30] Charlton M. Obesity, hyperlipidemia, and metabolic syndrome. Liver Transpl. 2009;15 Suppl 2:S83-9. doi: 10.1002/lt.21914 19877024

[31] Katsimardou A, Imprialos K, Stavropoulos K, Sachinidis A, Doumas M, Athyros V. Hypertension in Metabolic Syndrome: Novel Insights. Curr Hypertens Rev. 2020;16(1):12–8. doi: 10.2174/1573402115666190415161813 30987573

[32] Davis TME. Diabetes and metabolic dysfunction-associated fatty liver disease. Metabolism. 2021;123:154868. doi: 10.1016/j.metabol.2021.154868 34400217

[33] Gurevitz C, Rosenson RS. Metabolic Dysfunction-Associated Steatotic Liver Disease, Hypertriglyceridemia and Cardiovascular Risk. Eur J Prev Cardiol. 2024:zwae388. doi: 10.1093/eurjpc/zwae388 39656826

[34] Chen C, Zhang W, Yan G, Tang C. Identifying metabolic dysfunction-associated steatotic liver disease in patients with hypertension and pre-hypertension: An interpretable machine learning approach. Digit Health. 2024;10:20552076241233135. doi: 10.1177/20552076241233135 38389508 PMC10883118

[35] Qi X, Li J, Caussy C, Teng G-J, Loomba R. Epidemiology, screening, and co-management of type 2 diabetes mellitus and metabolic dysfunction-associated steatotic liver disease. Hepatology. 2024:10.1097/HEP.0000000000000913. doi: 10.1097/HEP.0000000000000913 38722246 PMC12904236

[36] Amangurbanova M, Huang DQ, Noureddin N, Tesfai K, Bettencourt R, Siddiqi H, et al. A Prospective Study on the Prevalence of MASLD in Patients With Type 2 Diabetes and Hyperferritinaemia. Aliment Pharmacol Ther. 2025;61(3):456–64. doi: 10.1111/apt.18377 39499168

[37] Wang Q, Zhu M, Li H, Chen P, Wang M, Gu L, et al. Hyperferritinemia Correlates to Metabolic Dysregulation and Steatosis in Chinese Biopsy-Proven Nonalcoholic Fatty Liver Disease Patients. Diabetes Metab Syndr Obes. 2022;15:1543–52. doi: 10.2147/DMSO.S361187 35607608 PMC9124058

[38] Pietrangelo A. Hereditary hemochromatosis: pathogenesis, diagnosis, and treatment. Gastroenterology. 2010;139(2):393–408, 408.e1-2. doi: 10.1053/j.gastro.2010.06.013 20542038

[39] Suresh D, Li A, Miller MJ, Wijarnpreecha K, Chen VL. Associations between metabolic hyperferritinaemia, fibrosis-promoting alleles and clinical outcomes in steatotic liver disease. Liver Int. 2024;44(2):389–98. doi: 10.1111/liv.15787 37971775 PMC10872664

[40] Bansal SK, Bansal MB. Pathogenesis of MASLD and MASH - role of insulin resistance and lipotoxicity. Aliment Pharmacol Ther. 2024;59 Suppl 1:S10–22. doi: 10.1111/apt.17930 38451123

[41] Liu C, Li N, Sheng D, Shao Y, Qiu L, Shen C, et al. Increased visceral fat area to skeletal muscle mass ratio is positively associated with the risk of metabolic dysfunction-associated steatotic liver disease in a Chinese population. Lipids Health Dis. 2024;23(1):104. doi: 10.1186/s12944-024-02100-5 38616253 PMC11016208

[42] Danisi JM, Fernandez-Mendoza J, Vgontzas AN, Calhoun SL, He F, Liao D, et al. Association of visceral adiposity and systemic inflammation with sleep disordered breathing in normal weight, never obese adolescents. Sleep Med. 2020;69:103–8. doi: 10.1016/j.sleep.2020.01.011 32062036 PMC7200279

[43] Panizza CE, Wong MC, Kelly N, Liu YE, Shvetsov YB, Lowe DA, et al. Diet Quality and Visceral Adiposity among a Multiethnic Population of Young, Middle, and Older Aged Adults. Curr Dev Nutr. 2020;4(6):nzaa090. doi: 10.1093/cdn/nzaa090 33959689 PMC8082229

[44] Mignini I, Galasso L, Piccirilli G, Calvez V, Termite F, Esposto G, et al. Interplay of Oxidative Stress, Gut Microbiota, and Nicotine in Metabolic-Associated Steatotic Liver Disease (MASLD). Antioxidants (Basel). 2024;13(12):1532. doi: 10.3390/antiox13121532 39765860 PMC11727446

[45] Aleksandrova K, Koelman L, Rodrigues CE. Dietary patterns and biomarkers of oxidative stress and inflammation: A systematic review of observational and intervention studies. Redox Biol. 2021;42:101869. doi: 10.1016/j.redox.2021.101869 33541846 PMC8113044

[46] Liu W, Wang J, Wang M, Hou H, Ding X, Ma L, et al. Oxidative Stress Factors Mediate the Association Between Life’s Essential 8 and Accelerated Phenotypic Aging: NHANES 2005-2018. J Gerontol A Biol Sci Med Sci. 2024;79(1):glad240. doi: 10.1093/gerona/glad240 37813096

[47] Adolph TE, Tilg H. Western diets and chronic diseases. Nat Med. 2024;30(8):2133–47. doi: 10.1038/s41591-024-03165-6 39085420

[48] Haigh L, Kirk C, El Gendy K, Gallacher J, Errington L, Mathers JC, et al. The effectiveness and acceptability of Mediterranean diet and calorie restriction in non-alcoholic fatty liver disease (NAFLD): A systematic review and meta-analysis. Clin Nutr. 2022;41(9):1913–31. doi: 10.1016/j.clnu.2022.06.037 35947894

