# Supplementary figures and images for: Exploring the association between dietary indices and metabolic dysfunction-associated steatotic liver disease: Mediation analysis and evidence from NHANES
Source: PLoS One. 2025 Apr 17;20(4):e0321251. doi: 10.1371/journal.pone.0321251 (PMC12005519; doi:10.1371/journal.pone.0321251)

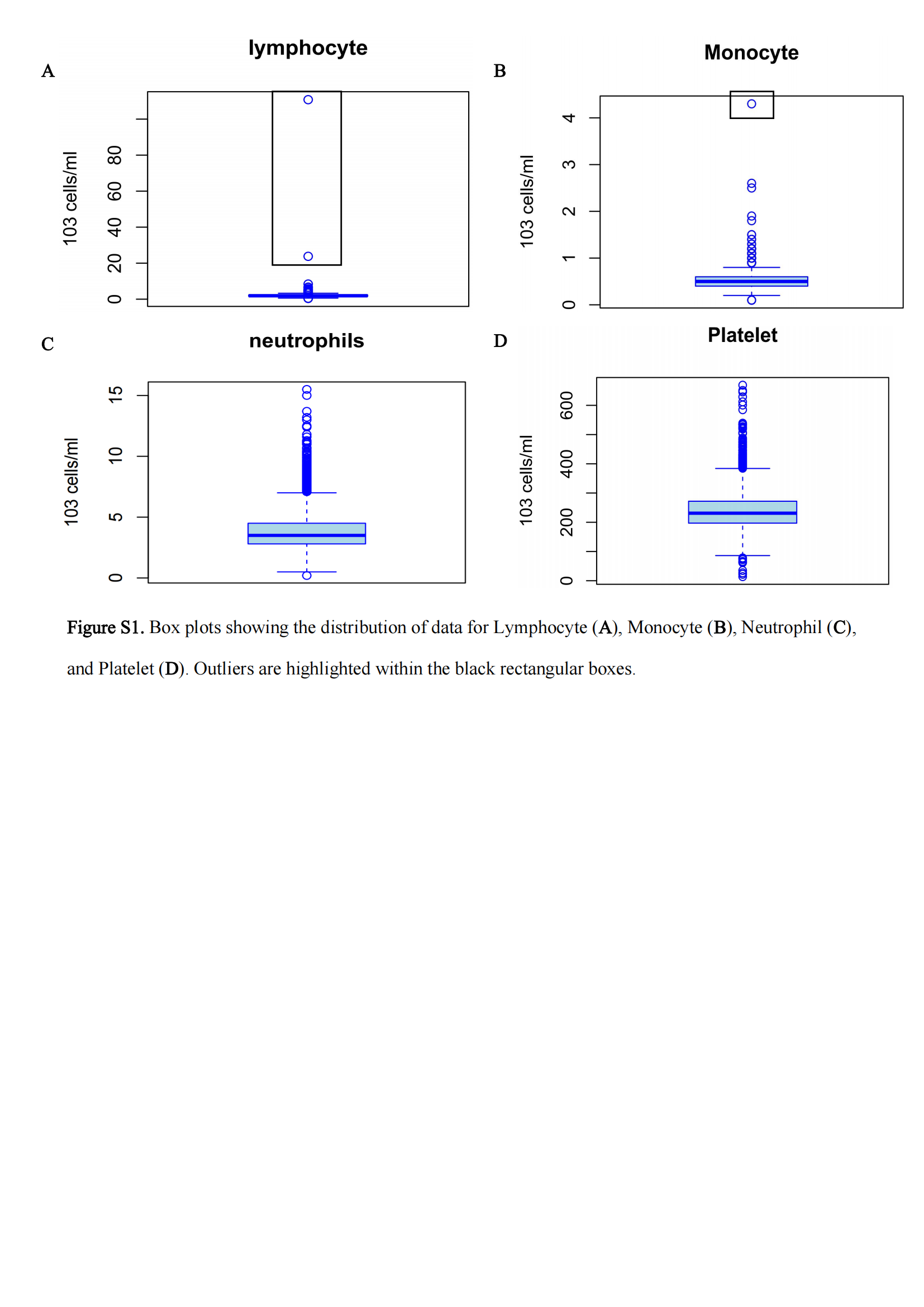

Supplement: S1 Fig — Outliers are highlighted within the black rectangular boxes. (TIF) [file pone.0321251.s001.tif]
