# Supplementary material for: Exploring the association between dietary indices and metabolic dysfunction-associated steatotic liver disease: Mediation analysis and evidence from NHANES
Source: PLoS One. 2025 Apr 17;20(4):e0321251. doi: 10.1371/journal.pone.0321251 (PMC12005519; doi:10.1371/journal.pone.0321251)
Supplement: S2 Table — (DOCX) [file pone.0321251.s003.docx]

| **Variables** | **Division details** | **Corresponding range (mg)** | **Frequency** | **Percentage** |
| --- | --- | --- | --- | --- |
| **[Healthy eating index](https://www.sciencedirect.com/science/article/pii/S2405457723001377)** | Q1 | [18.134, 44.469] | 1593 | 25% |
|  | Q2 | (44.469, 54.053] | 1592 | 25% |
|  | Q3 | (54.053, 64.492] | 1592 | 25% |
|  | Q4 | (64.492, 96.457] | 1592 | 25% |
| **Energy density dietary inflammatory index** | Q1 | [-3.072, -0.040] | 1593 | 25% |
|  | Q2 | (-0.040,0.679] | 1592 | 25% |
|  | Q3 | (0.679,1.566] | 1592 | 25% |
|  | Q4 | (1.566,53.009] | 1592 | 25% |
| **[Composite dietary antioxidant index](https://link.springer.com/article/10.1007/s00774-023-01438-7)** | Q1 | [-7.711, -1.926] | 1593 | 25% |
|  | Q2 | (-1.926, 0.350] | 1593 | 25% |
|  | Q3 | (0.350, 3.092] | 1593 | 25% |
|  | Q4 | (3.092, 122.701] | 1590 | 25% |

**Table S2**. Details of dietary indexs division.
