# Supplementary material for: Exploring the association between dietary indices and metabolic dysfunction-associated steatotic liver disease: Mediation analysis and evidence from NHANES
Source: PLoS One. 2025 Apr 17;20(4):e0321251. doi: 10.1371/journal.pone.0321251 (PMC12005519; doi:10.1371/journal.pone.0321251)
Supplement: S3 Table — Unadjusted model: non-adjusted model. Adjust 1: Adjust for age, sex, race. Adjust 2: Adjust for age, sex, race, body mass index, education levels, marital status, e density lipoprotein. Abbreviations: HEI, healthy eating index; CI, confidence interval. (DOCX) [file pone.0321251.s004.docx]

**Table S3*.*** Adjusted association of dietary indexs with MASLD for sensitivity analysis.

| **Exposure** | **Unadjusted model** | **Adjust 1** | **Adjust 2** |
| --- | --- | --- | --- |
|  | Odds ratio (95% CI) associated with MASLD | | |
| **HEI (continuous)** | 0.99 (0.98, 0.99); **< 0.001** | 0.98 (0.97, 0.98); **< 0.001** | 0.98 (0.98, 0.99); **< 0.001** |
| **Quartile of HEI** |  |  |  |
| Q1 | 1 (Ref) | 1 (Ref) | 1 (Ref) |
| Q2 | 0.97 (0.85, 1.10); 0.610 | 0.86 (0.75, 0.97); **0.020** | 0.93 (0.74, 1.16); 0.523 |
| Q3 | 0.77 (0.67, 1.89); **< 0.001** | 0.63 (0.55, 0.73); **< 0.001** | 0.79 (0.63, 0.99); **0.040** |
| Q4 | 0.59 (0.52, 0.68); **< 0.001** | 0.44 (0.39, 0.51); **< 0.001** | 0.52 (0.40, 0.69); **< 0.001** |
| *P* for trend | **< 0.001** | **< 0.001** | **< 0.001** |

Unadjusted model: Non-adjusted model.

Adjust 1: Adjust for age, sex, race.

Adjust 2: Adjust for age, sex, race, body mass index, education levels, marital status, smoking status, hyperlipidemia, hypertension, diabetes mellitus, triglyceride and high density lipoprotein.

**Abbreviations**: HEI, [healthy eating index](https://www.sciencedirect.com/science/article/pii/S2405457723001377); CI, confidence interval.
