# Supplementary material for: Exploring the association between dietary indices and metabolic dysfunction-associated steatotic liver disease: Mediation analysis and evidence from NHANES
Source: PLoS One. 2025 Apr 17;20(4):e0321251. doi: 10.1371/journal.pone.0321251 (PMC12005519; doi:10.1371/journal.pone.0321251)
Supplement: S4 Table — Abbreviations: HEI, healthy eating index; CI, confidence interval. (DOCX) [file pone.0321251.s005.docx]

**Table S4*.*** Nonlinear *P*-values of HEI and MASLD at different knots.

| **HEI knots** | **Nonlinear P-values** |
| --- | --- |
| 3 | 0.975 |
| 4 | 0.399 |
| 5 | 0.175 |
| 6 | 0.363 |
| 7 | 0.105 |
| 8 | 0.097 |

**Abbreviations**: HEI, [healthy eating index](https://www.sciencedirect.com/science/article/pii/S2405457723001377); CI, confidence interval.
