# Supplementary material for: Exploring the association between dietary indices and metabolic dysfunction-associated steatotic liver disease: Mediation analysis and evidence from NHANES
Source: PLoS One. 2025 Apr 17;20(4):e0321251. doi: 10.1371/journal.pone.0321251 (PMC12005519; doi:10.1371/journal.pone.0321251)
Supplement: S5 Table — Unadjusted model: non-adjusted model. Adjust 1: Adjust for age, sex, race. Adjust 2: Adjust for age, sex, race, body mass index, poverty income ratio, education levels, marital status, smoking status, alcohol consumption, hyperlipidemia, hypertension, diabetes mellitus, triglyceride, high density lipoprotein and PA total MET. Abbreviations: HEI, healthy eating index; METS-IR, metabolic score for insulin resistance; HOMA-IR, homeostatic model assessment of insulin resistance; SII, systemic immune-inflammation index; SIRI, systemic inflammation response index; BRI, body roundness index; ABSI, a body shape index; GGT, serum gamma- glutamyltransferase; CI, confidence interval. (DOCX) [file pone.0321251.s006.docx]

**Table S5*.*** Relationship between HEI and potential mediators in different models.

| **Outcomes** | **Unadjusted model** | | **Adjust 1** | | **Adjust 2** |
| --- | --- | --- | --- | --- | --- |
|  | β (95% CI ) associated with potential mediating variables | | | | |
| **METS-IR** | -0.12 (-0.14, -0.09); **< 0.001** | -0.13 (-0.15, -0.10); **< 0.001** | | -0.02 (-0.03, -0.01); **0.004** | |
| **HOMA-IR** | -0.02 (-0.04, -0.01); **< 0.001** | -0.03 (-0.04, -0.02); **< 0.001** | | -0.01 (-0.02, 0.00); **0.04** | |
| **SII** | -1.78 (-2.35, -1.20); **< 0.001** | -2.62 ( -3.25, -2.00); **< 0.001** | | -1.69 (-2.30, -1.08); **< 0.001** | |
| **SIRI** | 0 (-0.01, 0.00); **<0.001** | -0.01 (-0.01, 0.00); **< 0.001** | | 0 (0.00, 0.00); **< 0.001** | |
| **BRI** | -0.01 (-0.02, -0.01); **< 0.001** | -0.02 (-0.03, -0.02); **< 0.001** | | -0.01 (-0.01, 0.00); **< 0.001** | |
| **ABSI** | 0 (0.00, 0.00); **0.04** | 0 (0.00, 0.00); **< 0.001** | | 0 (0.00, 0.00); **0.01** | |
| **GGT** | -0.08 (-0.12, -0.03); **< 0.001** | -0.08 (-0.12, -0.04); **< 0.001** | | -0.03 (-0.08, 0.02); 0.21 | |
| **Bilirubin** | 0 (0.00, 0.00); **< 0.001** | 0 (0.00, 0.00); **< 0.001** | | 0 (0.00, 0.00); **0.01** | |
| **Uric acid** | 0 (-0.01, 0.00); **0.03** | 0 (-0.01, 0.00); 0.01 | | 0 ( 0.00, 0.00); 0.66 | |

Unadjusted model: non-adjusted model.

Adjust 1: Adjust for age, sex, race.

Adjust 2: Adjust for age, sex, race, body mass index, poverty income ratio, education levels, marital status, smoking status, alcohol consumption, hyperlipidemia, hypertension, diabetes mellitus, triglyceride, high density lipoprotein and PA total MET.

**Abbreviations**: HEI, [healthy eating index](https://www.sciencedirect.com/science/article/pii/S2405457723001377); METS-IR, [metabolic score for insulin resistance;](https://link.springer.com/article/10.1186/s12933-024-02334-8) HOMA-IR, homeostatic model assessment of insulin resistance; SII, systemic immune-inflammation index; SIRI, systemic inflammation response index; BRI, body roundness index; ABSI, [a body shape index](https://onlinelibrary.wiley.com/doi/abs/10.1002/pros.24698); GGT, serum gamma- glutamyltransferase; CI, confidence interval.
