# Supplementary material for: Exploring the association between dietary indices and metabolic dysfunction-associated steatotic liver disease: Mediation analysis and evidence from NHANES
Source: PLoS One. 2025 Apr 17;20(4):e0321251. doi: 10.1371/journal.pone.0321251 (PMC12005519; doi:10.1371/journal.pone.0321251)
Supplement: S6 Table — Unadjusted model: non-adjusted model. Adjust 1: Adjust for age, sex, race. Adjust 2: Adjust for age, sex, race, body mass index, poverty income ratio, education levels, marital status, smoking status, alcohol consumption, hyperlipidemia, hypertension, diabetes mellitus, triglyceride, high density lipoprotein and PA total MET. Abbreviations: HEI, healthy eating index; METS-IR, metabolic score for insulin resistance; HOMA-IR, homeostatic model assessment of insulin resistance; SII, systemic immune-inflammation index; SIRI, systemic inflammation response index; BRI, body roundness index; ABSI, a body shape index; GGT, serum gamma- glutamyltransferase; CI, confidence interval. * To address the extreme OR values observed during the initial analysis, we scaled the ABSI values by multiplying them by 100. This transformation ensured that the variable was within a more interpretable and computationally stable range, without affecting the underlying associations. After this adjustment, the logistic regression model yielded reasonable and reliable OR estimates. (DOCX) [file pone.0321251.s007.docx]

**Table S6*.*** Relationship between HEI and potential mediators with MASLD in different models.

| **Exposure** | | **Unadjusted model** | **Adjust 1** | **Adjust 2** |
| --- | --- | --- | --- | --- |
|  |  | Odds ratio (95% CI) associated with MASLD | | |
| **Exposure 1** | **HEI** | 1.00 (1.00, 1.01); 0.39 | 0.99 (0.98, 1.00); **0.04** | 0.99 (0.98, 1.00); **0.01** |
|  | **METS-IR** | 1.47 (1.43, 1.50); **< 0.001** | 1.52 (1.48, 1.57); **< 0.001** | 1.59 (1.50, 1.69); **< 0.001** |
| **Exposure 2** | **HEI** | 0.99 (0.98, 1.00); **0.02** | 0.98 (0.98, 0.99); **< 0.001** | 0.99 (0.98, 1.00); **0.03** |
|  | **HOMA-IR** | 3.28 (2.93, 3.68); **< 0.001** | 3.37 (2.99, 3.80); **< 0.001** | 2.46 (2.14, 2.82); **< 0.001** |
| **Exposure 3** | **HEI** | 0.98 (0.98, 0.99); **< 0.001** | 0.98 (0.97, 0.98); **< 0.001** | 0.98 (0.97, 0.99); **< 0.001** |
|  | **SII** | 1.00 (1.00, 1.00); **< 0.001** | 1.00 (1.00, 1.00); **< 0.001** | 1.00 (1.00, 1.00); 0.07 |
| **Exposure 4** | **HEI** | 0.98 (0.98, 0.99); **< 0.001** | 0.98 (0.97, 0.98); **< 0.001** | 0.98 (0.97, 0.99); **< 0.001** |
|  | **SIRI** | 1.34 (1.22, 1.48); **< 0.001** | 1.17 (1.06, 1.29); **0.002** | 1.09 (0.96, 1.25); 0.17 |
| **Exposure 5** | **HEI** | 0.98 (0.97, 0.99); **< 0.001** | 0.99 (0.98, 1.00); **0.01** | 0.99 (0.98, 1.00); 0.07 |
|  | **BRI** | 4.67 (4.22, 5.16); **< 0.001** | 6.62 (5.80, 7.56); **< 0.001** | 4.89 (4.05, 5.91); **< 0.001** |
| **Exposure 6** | **HEI** | 0.98 (0.98, 0.99); **< 0.001** | 0.98 (0.97, 0.98); **< 0.001** | 0.98 (0.97, 0.99); **< 0.001** |
|  | **ABSI*** | 3.77 (3.11, 4.56); **< 0.001** | 2.49 (2.00, 3.09); **< 0.001** | 5.23 (3.85, 7.11); **< 0.001** |
| **Exposure 7** | **HEI** | 0.98 (0.98, 0.99); **< 0.001** | 0.98 (0.97, 0.98); **< 0.001** | 0.98 (0.98, 0.99); **< 0.001** |
|  | **GGT** | 1.05 (1.04, 1.06); **< 0.001** | 1.04 (1.03, 1.05); **< 0.001** | 1.06 (1.04, 1.07); **< 0.001** |
| **Exposure 8** | **HEI** | 0.98 (0.98, 0.99); **< 0.001** | 0.98 (0.97, 0.98); **< 0.001** | 0.98 (0.97, 0.98); **< 0.001** |
|  | **Bilirubin** | 0.77 (0.62, 0.96); **0.02** | 0.59 (0.45, 0.76); **< 0.001** | 1.08 (0.70, 1.66); 0.72 |
| **Exposure 9** | **HEI** | 0.98 (0.98, 0.99); **< 0.001** | 0.97 (0.97, 0.98); **< 0.001** | 0.98 (0.97, 0.99); **< 0.001** |
|  | **Uric acid** | 1.70 (1.61, 1.81); **< 0.001** | 1.73 (1.60, 1.86); **< 0.001** | 0.94 (0.66, 1.35); 0.74 |

Unadjusted model: non-adjusted model.

Adjust 1: Adjust for age, sex, race.

Adjust 2: Adjust for age, sex, race, body mass index, poverty income ratio, education levels, marital status, smoking status, alcohol consumption, hyperlipidemia, hypertension, diabetes mellitus, triglyceride, high density lipoprotein and PA total MET.

**Abbreviations**: HEI, [healthy eating index](https://www.sciencedirect.com/science/article/pii/S2405457723001377); METS-IR, [metabolic score for insulin resistance;](https://link.springer.com/article/10.1186/s12933-024-02334-8) HOMA-IR, homeostatic model assessment of insulin resistance; SII, systemic immune-inflammation index; SIRI, systemic inflammation response index; BRI, body roundness index; ABSI, [a body shape index](https://onlinelibrary.wiley.com/doi/abs/10.1002/pros.24698); GGT, serum gamma- glutamyltransferase; CI, confidence interval.

* To address the extreme OR values observed during the initial analysis, we scaled the ABSI values by multiplying them by 100. This transformation ensured that the variable was within a more interpretable and computationally stable range, without affecting the underlying associations. After this adjustment, the logistic regression model yielded reasonable and reliable OR estimates.
